# Mapping the Application of Welding Technologies in Remanufacturing of Molds and Dies

**DOI:** 10.3390/ma19163459

**Published:** 2026-08-14

**Authors:** Marina Grabarski, Bruno Bellini Medeiros, Giuseppe Pintaude

**Affiliations:** 1 Instituto Federal do Paraná campus Paranaguá, Eixo de Controle e Processos Industriais, Rua Antonio Carlos Rodrigues, Porto Seguro 83215-750, Brazil; marina.grabarski@ifpr.edu.br; 2 Academic Department of Mechanics, Universidade Tecnológica Federal do Paraná, Campus Curitiba, R. Dep. Heitor A. Furtado, 5000, Curitiba 81280-340, Brazil; brunomedeiros@professores.utfpr.edu.br

**Keywords:** plasma welding, PTAW, mold, die, remanufacturing, repair, AISI H13 steel

## Abstract

**Highlights:**

- Laser-based processes are the most employed to repair molds and dies.
- AISI H13 steel is the most used material as a metal filler for repairing molds and dies.
- PTAW is almost nil for tool repair, probably due to relevant initial current costs.
- PTAW is a potential technique to repair large molds or interior parts.
- A relevant gap exists in the performance data of repaired tools.

**Abstract:**

This review article is motivated by the possibility of applying plasma transferred arc welding (PTAW) for repairing molds and dies. To amplify this purpose, a bibliometric analysis was conducted using the Web of Science platform. More articles were needed to extend the understanding in more detail about the manufacturing of repairs. Around 64% of the manuscripts analyzed used laser sources, while around 34% were based on electric arc processes. The most common material seen as a substrate, and even as a metal addition, is the AISI H13 steel. Surprisingly, few studies have focused on real components, and there is little information about the performance of repaired tools. Moreover, there are gaps in the resulting microstructures and their manipulation according to the process variables. The employment of PTAW for repairing molds and dies is scarce, making it very difficult to compare the final performance among all available processes, although we could describe the relevant potential of this technique for large mold surfaces or interior repair.

## 1. Introduction

Forming and molding processes are responsible for manufacturing a wide range of components, particularly in the automotive, aerospace, construction, and electronics industries. These processes are characterized by high productivity for standardized parts and low material waste. Actions aimed at improving efficiency and reducing the environmental impacts of production are crucial for the sector’s growth [1]. It is estimated that up to 30% of a component’s manufacturing cost can be attributed to tooling [2]. The manufacturing of molds and dies is a complex and expensive process, making it essential to maximize their service life. These tools are subjected to extreme operating conditions and typically fail due to wear, mechanical fatigue, and plastic deformation. In hot-working processes, such as forging and stamping, thermal fatigue and oxidation also occur [3].

Once a tool fails, it must be replaced or repaired. Given that formed products require tight dimensional and geometric tolerances, any shape deviation in the tool caused by wear or misuse leads to production errors. Changes in tool design may also necessitate rework. In all such cases, remanufacturing may be considered.

Repairs can be performed by depositing material using a welding process, followed by machining. Repair-welded dies may have longer life than new dies [4]. However, before executing the repair, stages must be conducted to evaluate the tool’s remaining useful life. Chen et al. [5] discuss methods for assessing damage in a mold. It is not a straightforward task; inspection and numerical simulation are required to estimate damage evolution. Furthermore, an assessment of the economic viability of the repair must be performed [5].

Several welding processes can be used for this purpose, ranging from conventional arc processes such as gas metal arc welding (GMAW), gas tungsten arc welding (GTAW/TIG), and PTAW to high-focalized energy processes such as laser or electron beam welding. Each process presents its own characteristics regarding bead geometry, dilution, phase formation control, and cost. The availability of filler material is also a significant variable. Jhavar et al. discussed the selection of a welding process for mold repair, considering these and other variables [6].

In addition to cost reduction, mold repair can consume less energy and emit fewer pollutants than manufacturing a new tool. Morrow et al. [7] evaluated whether mold fabrication or repair via direct metal deposition (DMD) could be environmentally superior. It was observed that the repair process consumed less than half of the energy required to manufacture a new part. However, the authors noted that producing metallic powders consumes 25% more energy than conventional plate casting [7]. The environmental impact of metal powder production is also highlighted in the study by Stieberova et al. [8]. Through life cycle assessment (LCA), the researchers concluded that producing a mold via direct metal laser sintering (DMLS) can be 12% cheaper than conventional methods. They observed a reduction of approximately 50% in machining time and achieved mold optimization, resulting in more efficient cooling. The proposed manufacturing methodology proved to be both financially and environmentally advantageous [8]. The same issue is observed when the filler material is in wire format. Priarone et al. [9] studied the life cycle of AISI H13 steel inserts repaired using the wire arc additive manufacturing (WAAM) technique. This material is widely used for molds and dies but carries a high acquisition cost. The authors observed that the energy demand and carbon footprint per unit mass for wire production were 3.5 times higher than for producing a bulk part [9]. In all cases presented, even if the raw material acquisition cost is higher, repair remains more advantageous since the mass of material used is smaller than that required for a new part. Machining time is also lower, which is a major factor impacting energy consumption. Repairing a part to extend its service life is a core principle of the circular economy. Actions that promote the rational use of resources and decrease production’s environmental impact are essential for the sustainability of this sector [10].

With advancements in welding technology and the development of additive manufacturing processes, there has been an increase in studies applying these techniques to equipment recovery [11,12]. Kanishka and Acherjee [11] observed that molds and dies are the second most frequent type of component in studies utilizing additive manufacturing repair techniques, representing 13% of the total. They noted that more than half of the processes, regardless of the part type, use laser as the energy source, while only 11% utilize an electric arc.

In studies comparing welding processes for part repair, plasma transferred arc (PTA) stands out among arc processes [5,6,12,13]. Hirpara et al. [14] point out that PTAW offers a high deposition rate, better heat input control, and less distortion than conventional processes. Furthermore, it is cheaper and easier to operate than high-focalized energy techniques. The process appears capable of processing any type of material while allowing alloy customization, as it uses raw materials in powder form [14]. However, despite these advantages, the process is not widely used in part repair. Of the 44 studies evaluated by Aimin et al. regarding part repair, only three utilized plasma welding techniques [12]. Of the 10 studies highlighted in the review by Kanishka and Acherjee [11] that use arc additive manufacturing processes, only one focuses on plasma welding.

This work aims to conduct a literature review and a bibliometric analysis of repair processes for molds and dies. The focus will be on understanding whether the plasma welding technique is utilized for this purpose and how it can be applied. An evaluation of the materials used, both as base and filler metals, will also be performed.

## 2. Materials for Dies and Molds

There are four main types of molds and dies in industry. They are injection molds, stamping dies, forging dies, and die-casting dies [5].

Molds can be made from a large spectrum of materials, some of them exotic like papier-mache and plaster, but considering most of the processes involve high pressure and temperature, metals stand as the most used materials group. Molds for injection molding can be made from steel, aluminum, bronze, and zinc alloys, with steel being the predominant material. The steel alloys for injection molding molds have been developed for many years based on close cooperation between steel producers and customers. From this knowledge, the steel alloys for plastic molds were divided into 6 groups [15]:

1. Nitriding steels (AISI 4130) preferred for extruders; only used in molds in special cases.
2. Case-hardening steels (AISI 5120; 9310).
3. Pre-hardened steels (AISI P20, 420).
4. Through-hardening steels (AISI H11; D2; O2; 6F7 L6 mod).
5. Corrosion-resistant steels (AISI 420).
6. Precipitation hardening steels (AISI Maraging 300).

The most commonly used steel alloys used to produce molds and tools for injection molding, and their applications and weldability, are presented in Table 1.

Stamping die materials depend on the stamping process, the material that was being formed, and the quantity of parts produced. For blanking and piercing dies up to 1000 parts (AISI A2, O1), 10,000 parts (AISI A2, D2), 1,000,000 parts (AISI D2, D4, A11), and 10,000,000 parts (Carbide), steel grades as materials to be formed are considered. For pressing forming dies up to 1000 parts (AISI 4140), 10,000 parts (AISI 4140, D2), 100,000 parts (AISI 4140, O1, A2, D2), and 1,000,000 parts (AISI A2, D2, nitrided D2) are considered. Deep-drawing die materials selection depends beyond the already commented variables of the die components named punch or blankholder, but in general up to 10,000 parts (carburized AISI 4140, W1), 100,000 parts to punch (W1, carburized S1), 1,000,000 parts (A2, D2) for punch, and AISI W1 and O1 for blankholder independent of production range [16] are considered.

Hot forging die materials include hot work steels from the AISI H series, steel alloys from the AISI 4300 or 4100 series, and some low alloy steels such as AISI 6G, 6F2, and 6F3 [16]. Most common forging die materials are shown in Table 2 [17].

Die casting manufacturing process consists of the injection of non-ferrous alloys under high pressures and velocities in metallic reusable dies. Aluminum, magnesium, and zinc are the commonly processed alloys [18]. AISI H13 steel is the most popular material [19,20] and, together with AISI H11, is considered a typical material used for manufacturing aluminum die-casting dies [21].

## 3. Bibliometric Analysis

This review aimed to investigate the applicability of the plasma transferred arc welding process for the repair of molds and dies. A literature search was conducted on the Web of Science platform using the following keyword combination: (“repair” OR “remanufacturing”) AND (“mold” OR “die”) AND (“PTA” OR “PTAW” OR “plasma welding”).

Out of the four retrieved results, only two studies were specifically dedicated to the topic [22,23]. In the study by Jhavar et al., the use of micro-plasma transferred arc welding (µPTAW) was proposed for repairing molds and dies. The authors presented the technique as a viable alternative for small and medium-sized enterprises (SMEs) due to its lower investment cost compared to competing processes, such as laser beam or electron beam welding, while offering distinct advantages over conventional techniques. Single-bead deposition was performed using AISI P20 steel as the base metal and a filler wire of the same material. The filler wire had a diameter of only 300 µm, and the welding parameters were varied to determine the optimal combination. This optimized configuration was subsequently used to build a small wall of overlapping beads via additive manufacturing. The authors concluded that the deposits were executed satisfactorily, with negligible porosity. The process appears to be suitable for both parts’ recovery and additive manufacturing applications [22]. Despite the successful deposition, the work leaves some gaps. No considerations were made regarding the microstructure. The deposition did not occur on a real die. The tribological properties of the deposits were also not evaluated, making it impossible to assess the durability of the repair. The second study aimed to evaluate the effectiveness of PTAW-deposited hardfacing in the renovation of die-casting molds for aluminum automotive parts, with X38CrMoV5-1 (AISI H11) as the substrate. The authors concluded that all three coating powders (two variations in AISI M3:2 and a Ni-based alloy) can serve the purpose of renovating the mold surface [23].

As previously mentioned, the literature frequently highlights the benefits of the PTAW technique. This process appears to offer advantages comparable to high-energy beam processes but at a lower cost. Nevertheless, only two studies were found regarding the use of this technique for the recovery of the target components. Consequently, two further questions emerged: (1) which process is the most widely researched for this specific application, and (2) what types of components are typically recovered using the PTAW technique? All subsequent searches were also conducted on the Web of Science platform. While these results are understood to represent a significant portion of the published literature, some relevant studies may not have been retrieved due to indexing variations. Thus, the collected data constitutes a representative sample of the field.

The search for alternative processes was executed using the string: (“repair” OR “remanufacturing”) AND (“mold” OR “die”). By allowing the search terms to appear anywhere within the title, abstract, keywords, or text, the results yielded over 300 papers. An initial screening was performed by restricting the publication to engineering, environmental, and material sciences areas on the Web of Science page. Subsequently, the titles, keywords, and abstracts were analyzed. Texts were retained if they stated—at least in the abstract—that the evaluated process could be used for the repair of molds or dies. Only texts employing a welding method for restoration were selected. No restrictions were placed on the types of alloys processed. This assessment resulted in 106 papers.

**Figure 1:**
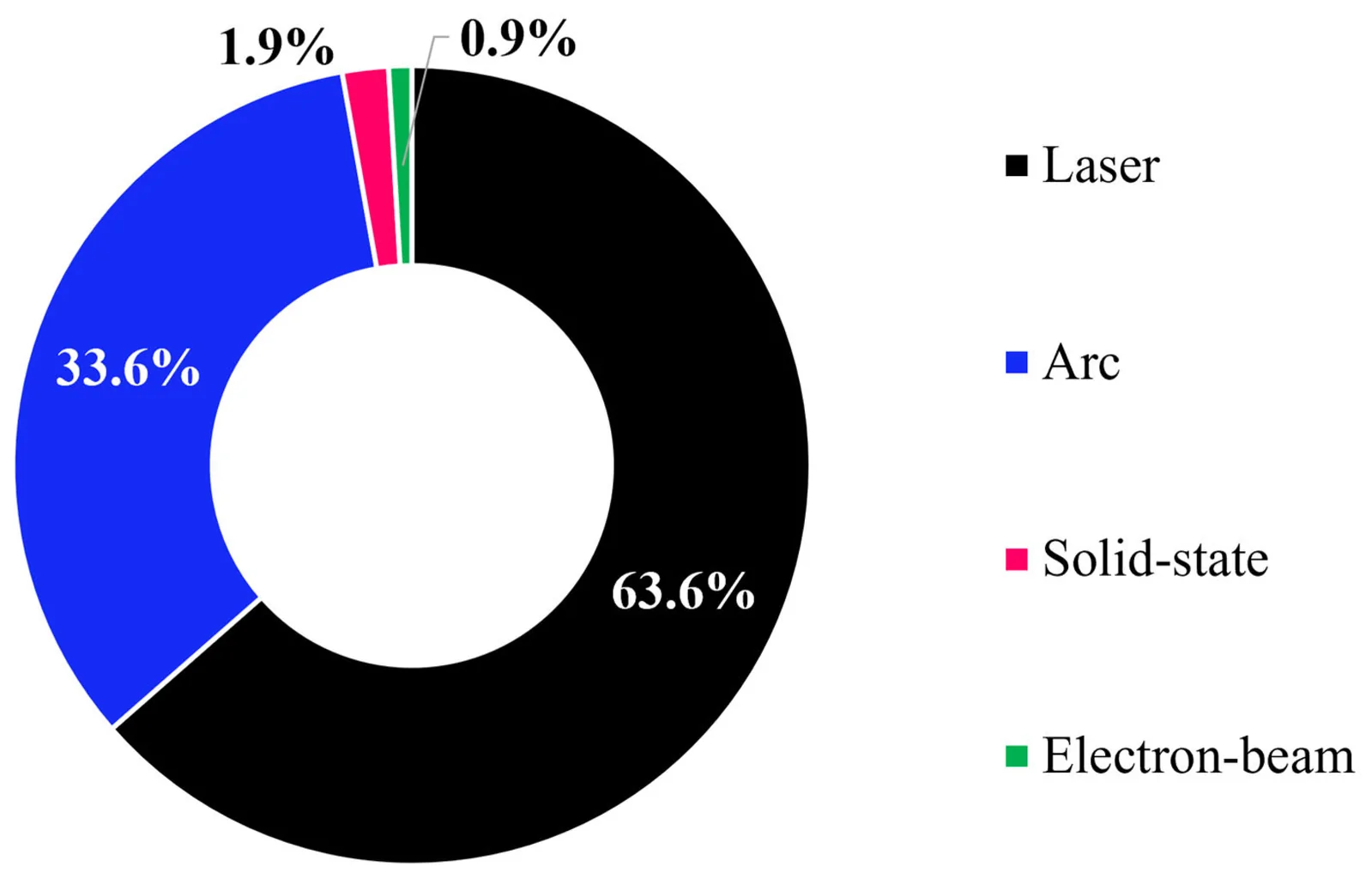
Classification of papers according to energy source.

The initial stage of text analysis was to classify the energy source utilized in the repair processes. The methods were categorized into laser, electron beam, and electric arc sources. A solid-state welding category was also established for processes that utilize mechanical energy to join materials. As illustrated in the chart in Figure 1, laser-based sources were the most prevalent, accounting for approximately 64% of the studies [7,24,25,26,27,28,29,30,31,32,33,34,35,36,37,38,39,40,41,42,43,44,45,46,47,48,49,50,51,52,53,54,55,56,57,58,59,60,61,62,63,64,65,66,67,68,69,70,71,72,73,74,75,76,77,78,79,80,81,82,83,84,85,86,87,88,89,90], followed by electric arc processes at nearly 34% [9,22,91,92,93,94,95,96,97,98,99,100,101,102,103,104,105,106,107,108,109,110,111,112,113,114,115,116,117,118,119,120,121,122,123]. These proportions are consistent with the results reported in the study by Kanishka and Acherjee [11]. Electron beam welding and solid-state joining processes were also identified, though with marginal representation [124,125,126].

**Figure 2:**
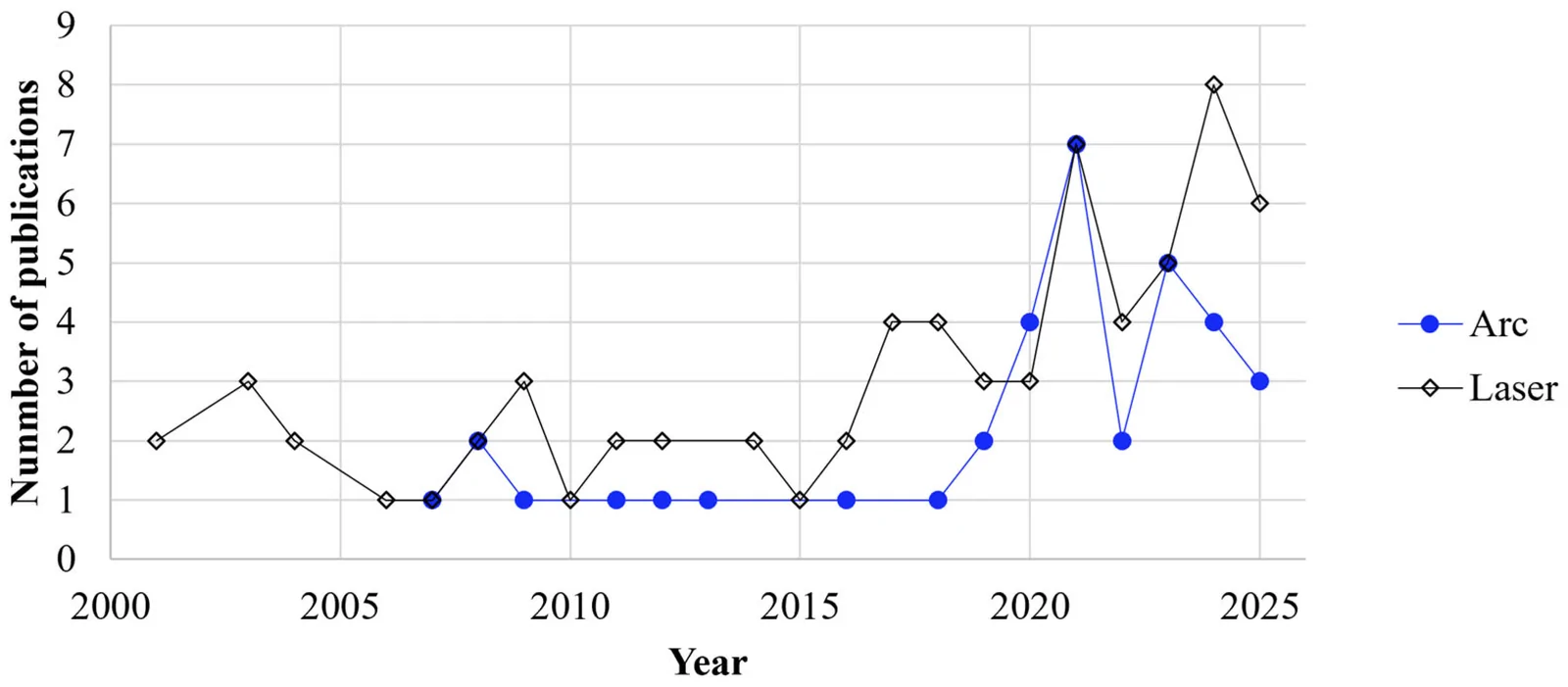
Number of publications on die repair over the years since 2000.

The selected papers were published from the year 2000 onward. As shown in Figure 2, the number of publications on arc-welding processes for die repair never exceeded that on laser-based processes at any point. Furthermore, a noticeable increase in the number of such publications is observed after 2017, demonstrating that this remains a highly relevant research topic.

**Figure 3:**
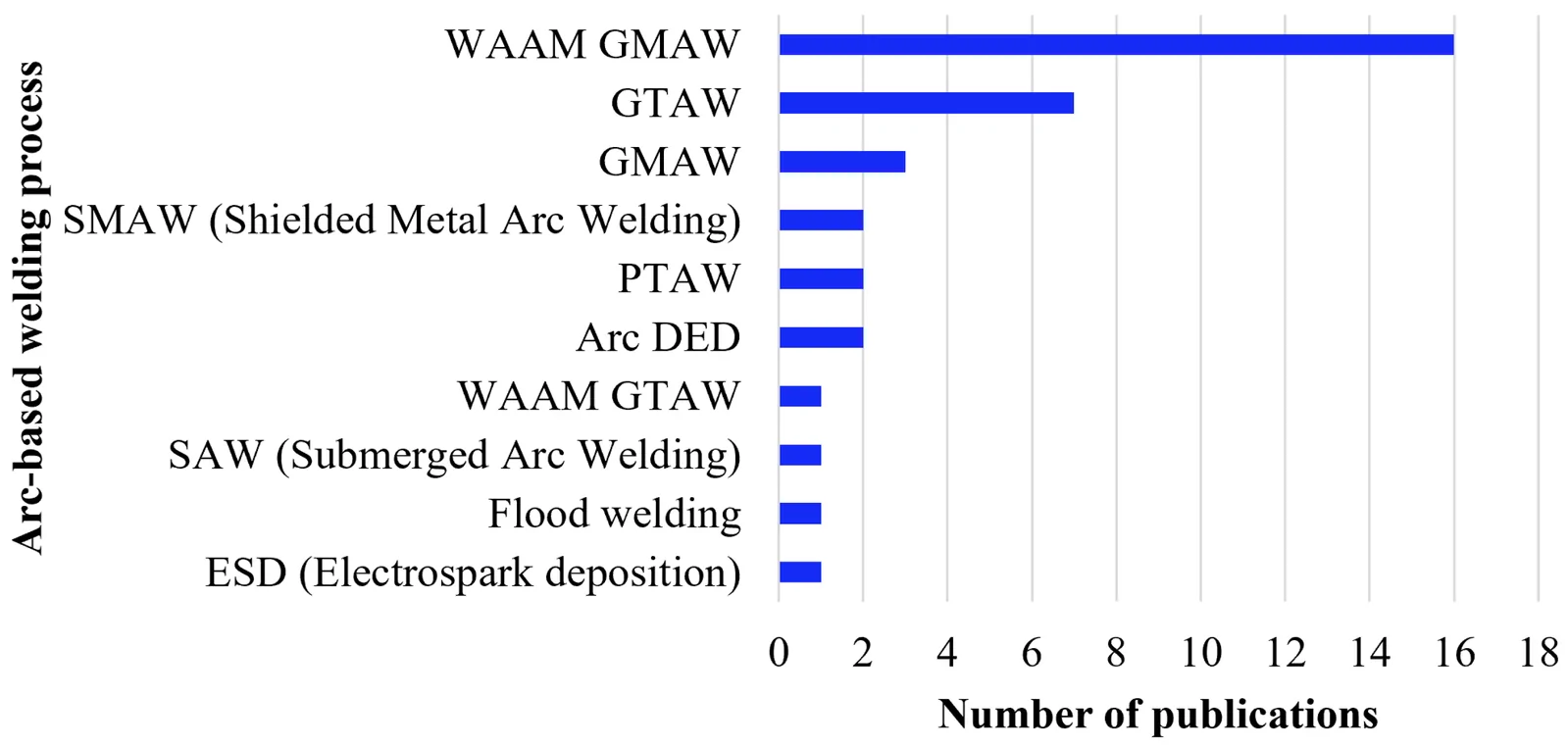
Number of publications per electric arc welding process used in die repair.

**Figure 4:**
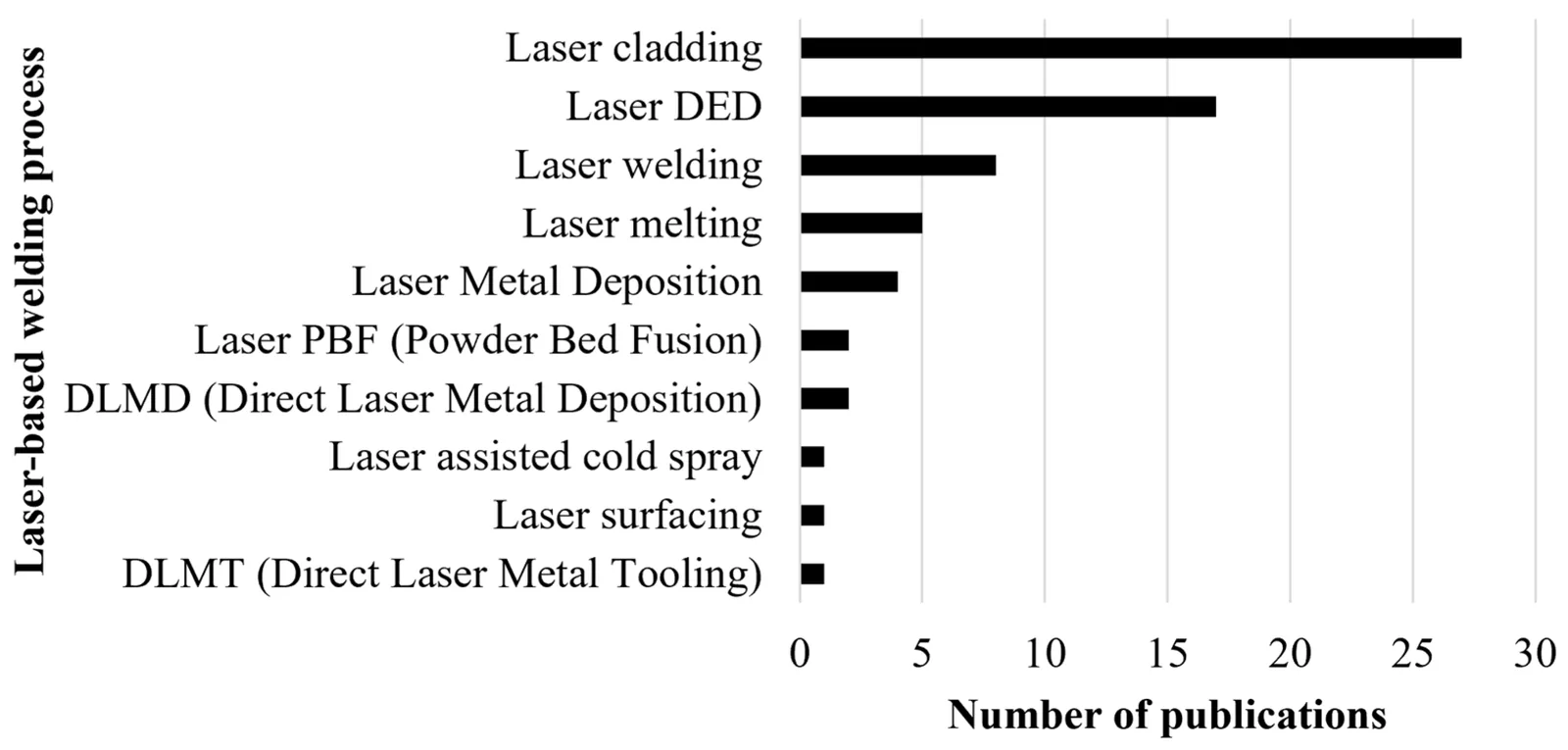
Number of publications per laser-based process used in die repair.

In the second stage of the analysis, the welding processes were classified. To avoid an excessive number of subcategories, several techniques were consolidated into broader groups, though the specific terminology utilized by the original authors was strictly maintained. Notably, several researchers emphasized that the repair techniques employed are classified within the domain of additive manufacturing. To preserve this distinction, the nomenclature was left unchanged, even where certain methods appear repetitive across the data. Figure 3 illustrates the processes utilizing an electric arc as an energy source, where wire arc additive manufacturing, typically employing GMAW equipment, emerged as the most frequently cited method. Among laser-based sources, laser cladding is the predominant technique, followed closely by laser-directed energy deposition (Laser-DED), as detailed in the distribution presented in Figure 4.

Among arc welding processes, GMAW equipment is the most widely used. This technique stands out for its deposition rate and low cost [111]. It is also one of the most common processes in industry and requires a low level of operator skill. Notably, slightly more than half of the studies utilize additive manufacturing techniques for deposition (WAAM and DED), which also indicates a trend in this sector.

All the laser processes shown in the chart in Figure 4 are similar. The laser source causes the material to melt, and filler material may be added. Laser cladding is generally associated with layer deposition for surface modification, whereas processes such as L-DED and laser metal deposition are more closely associated with multilayer deposition for three-dimensional fabrication [11]. However, for the repair of any component, the chosen welding process must allow for the deposition of multiple layers to fill defects.

**Figure 5:**
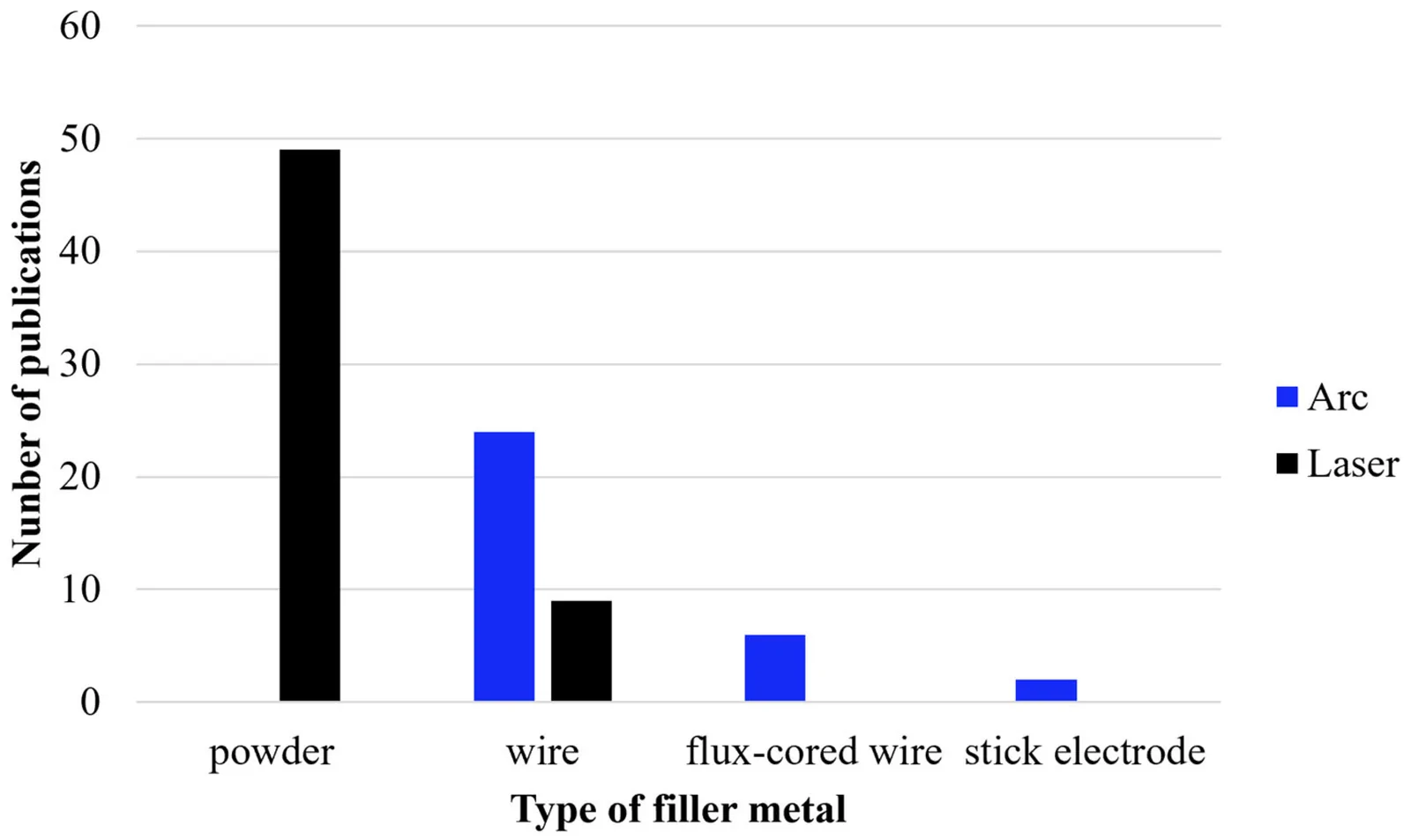
Types of filler material formats according to the process energy source used in die repair.

Another characteristic observed during the analysis was the type of filler metal employed, regarding both its physical format and chemical composition. Initially, concerning the format, powders and wires were identified as the most prevalent delivery methods. As illustrated in the chart in Figure 5, the types of filler material are categorized according to the energy source utilized. It is observed that laser-based processes predominantly utilize metallic powders. Conversely, arc processes do not employ powders, only utilizing wires and consumable electrodes.

Using filler metal in powder form offers greater freedom in selecting the chemical composition of the deposit [14]. It is possible to vary the percentage of an elemental powder or to add hard particles [43,65,70]. In contrast, processes that use wire-fed raw materials are constrained by chemical composition limitations, given the difficulty of producing wire in small batches. Chemical composition can also be a limiting factor in wire production; in the work by Jhavar, the wire used for the deposits had a lower carbon content. Consequently, the material exhibited higher ductility, enabling its manufacture. However, the resulting deposits displayed mechanical behavior different from that of the base material [22].

**Figure 6:**
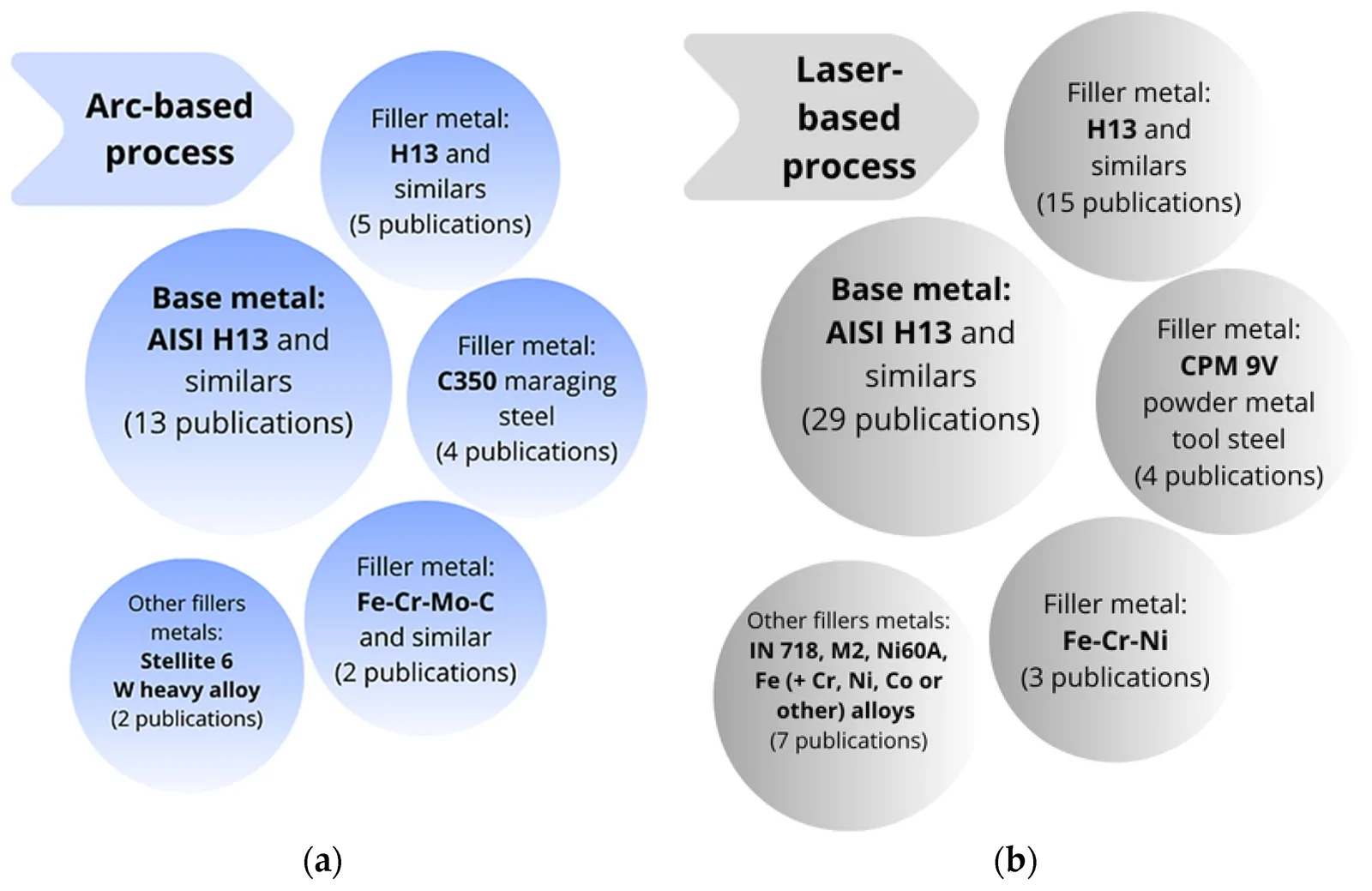
Chemical composition of filler materials utilized in the recovery of AISI H13 steel dies using electric arc (**a**) and laser (**b**) processes.

Regarding chemical composition, AISI H13 steel stands out as the most prevalent base metal, identified in 42 publications. For bibliometric purposes, similar or modified compositions were grouped into a single category. Figure 6 illustrates the chemical compositions of the filler materials used in studies involving AISI H13 steel, comparing electric arc processes (a) and laser-based processes (b). A greater variety of alloys is observed in laser-based processes, with several studies employing customized alloys to evaluate the influence of elemental content on repair quality [34,43,44,65,70,83]. In the next section, a more detailed discussion regarding the microstructure will be presented, based on the articles cited in Figure 6.

Among studies utilizing laser-based processes, AISI H11 and D2 alloys also emerge as significant base metals, featured in nine and four publications, respectively.

It is well-established that preheating can be fundamental for processing alloys with low weldability, such as tool steels, without the occurrence of cracking. In the evaluated studies, it was observed that preheating was conducted during welding in 37% of the publications utilizing arc processes, compared to only 9% for laser-based processes.

A search was conducted for articles addressing the recovery of any component type using plasma welding. The search string employed was: (“repair” OR “remanufacturing”) AND (“PTA” OR “PTAW” OR “plasma welding”). Out of the 44 initial results, only eight specifically addressed the topic of interest. These articles are presented in Table 3. It is noted that no specific pattern exists regarding the components remanufactured by this technique. Furthermore, in most studies, the repair was not performed on an actual part; instead, a bead was deposited onto a plate of the interested material. Consequently, in Table 3, these studies are classified as “bead on plate”. Only one research study applies plasma welding to the recovery of molds and dies [22]. This work was already detailed in this section.

Furthermore, no distinct pattern was identified regarding either the base or filler materials employed. While steel was predominantly utilized, titanium, nickel, and cobalt alloys were also identified. Among the eight studies evaluated, half used filler material in wire form [22,129,130,131], while the other half used powder [13,127,128,132]. As discussed in relation to Figure 5, the ability to process both filler forms is an advantage of the PTAW process. Notably, preheating was either not utilized or not explicitly mentioned in the literature. These findings suggest that the plasma process offers significant versatility regarding material compatibility and facilitates the use of powder as a filler. This enables the customization of alloys that are otherwise difficult to obtain in wire format; a capability previously noted in laser-based processes. Ultimately, it is observed that the plasma process is not conventionally utilized for the repair of molds and dies.

Finally, a search was conducted to identify reports of any PTAW deposits on substrates of materials used in die and mold manufacturing. The string (%PTA* and D2) OR (%PTA* and H13) OR (%PTA* and H11) OR (%PTA* and P20) OR (%PTA* and “tool steel”) returned 85 results in topic style (abstract, title, keywords), and after a manual screening, the number was reduced to 29 papers. AISI D2 alloy as substrate have been reported in [133,134,135,136,137,138,139,140,141,142], AISI P20 [22,129,143,144], AISI H13 [145,146,147], AISI H10 (X32CrMoV33) [148,149], Uddeholm Calmax [150,151], AISI M3:2 [152], AISI L6 (56NiCrMoV7) [153], AISI H11 (X38CrMoV5) [23,153], AISI O2 (90MnCrV8) [154], AISI 4140 [155,156], and gray cast iron [157,158]. From the results, one that stands out is AISI D2, holding 10 studies.

**Figure 7:**
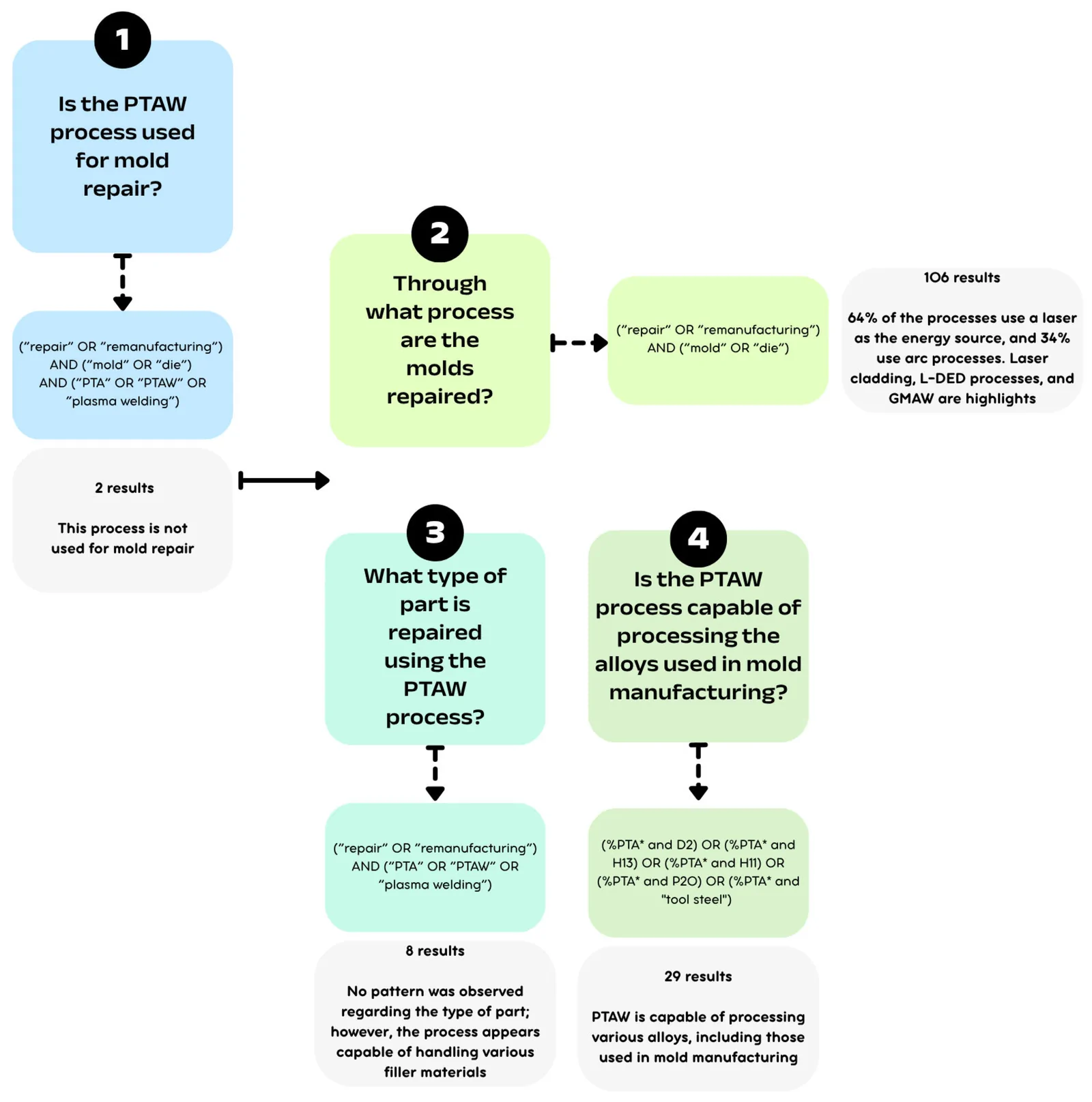
Summary of the four search queries used for the bibliometric analysis.

Figure 7 aims to summarize the questions that guided this Bibliometric Analysis. The initial question—highlighted by the first query—was to understand how the PTAW process was applied to mold repair. However, the lack of results prompted further questions: specifically, what process was used for mold repair? This led to a better understanding of the research scenario regarding this topic. With the aim of comparing the processes, further discussions based on the texts collected from query 2 will be presented in the next section.

Two additional lines of inquiry emerged in questions 3 and 4: has the PTAW been employed for the remanufacturing of other types of components? Do material-related processing constraints exist for the alloys used in mold and die manufacturing? As it imposes no restrictions on alloys and allows the use of filler materials in various forms, the PTAW process appears suitable for repairing metal parts. To better understand this process, the next section begins with some considerations regarding PTAW and comparisons with other processes. The criteria for selecting a process for mold repair will also be explored.

## 4. Die and Mold Repairing Processes: Comparing the Concurrent Alternatives

Laser, GTAW/TIG, and PTAW are among the most widely used processes for mold and die repair [5,159,160,161]. Despite PTAW being included in this group, some authors considered it a potential process but not widely used to repair [162]. Moreover, since the late 20th century, micro-welding techniques such as µ-PTAW and micro-gas tungsten arc welding (µ-GTAW) have gained attention in studies about repairing molds and dies [147]. Considering that these are the processes most relevant in the repair industry context, data were consolidated from review papers [6,162,163] for comparison purposes (see Table 4).

From these three review papers, the most significant evaluation criteria reported were picked: deposition rate, equipment portability, access and ability to repair complex geometries, metallurgical quality, post-weld heat treatment, cost, and setup time. This comparison shows the significance each author assigns to each criterion and how subjective that is, except for cost on TIG and laser processes and equipment portability on PTAW; on all other criteria, the authors do not agree with the score they attribute to that criterion. However, and maybe most importantly, they agree on whether a process is better or not on that specific criterion. Generally speaking, and as an example, all the authors agree that laser-based processes are the most costly, with higher setup time, low equipment portability, and deposition rate, and bad access and ability to repair complex geometries; on the other hand, they have the best metallurgical quality and low need for post-heat treatment.

Laser-based processes are characterized by a highly focused beam that heats a very small area, resulting in an accurate, distortion-free, and high-quality bead [162,163,164,165]. The heat-affected zone (HAZ) is small due to a low heat input, so the welds exhibit good metallurgical quality and likely do not require pre- or post-heat treatment [162]. The drawbacks are the relatively high cost, the low equipment portability and deposition rate, difficulty in accessing intricate geometries, and a more complex equipment and control set-up.

PTAW and GTAW are very similar [163,165], with PTAW being an advance of the GTAW process [5]. Both are known to have a stable arc, although PTAW is better in this regard due to the more constrained orifice, which makes it less vulnerable to arc length variation and delivers higher penetration [5]. PTAW has a lower heat input with a smaller heat-affected zone [162] at the detriment of deposition rate, whereas GTAW has higher performance, as two works agree [162,163], although Jhavar et al. [6] put them as the same in this criterion.

The µ-PTAW and µ-GTAW processes are evolutions of PTAW and GTAW, having a welding power supply, usually an inverter, able to control small currents with a precision of 0.1 A, comprising a 100 µm weld area using fillers with a few microns in diameter [6]. These features make these microwelding processes close to laser-based processes at lower cost in terms of precision, metallurgical properties, and heat treatment requirements, while keeping good equipment portability and access to intricate geometries inherited from the conventional processes, despite having the lowest deposition rate among all processes compared.

An intriguing issue is the scarcity of investigations regarding PTAW for remanufacturing. Some hypotheses can be raised, in view of more detailed aspects of costs. A clue was found in reference [165]. There, the following quote “for each laser welding machine existent in repair shop there is an average of 6 TIG welding machines,” helps to understand the scenario. Therefore, it is obvious that the volume of accessories, consumables, specific raw materials, and other supply issues is much higher than all other processes. As PTAW concurs directly with GTAW/TIG, a reasonable hypothesis is that the initial cost for installing a PTAW platform is higher than that of a GTAW one, even though in Table 4 there is a rough similarity in terms of low cost between GTAW and PTAW. In fact, there is no consensus on the low cost of the PTAW technique.

**Figure 8:**
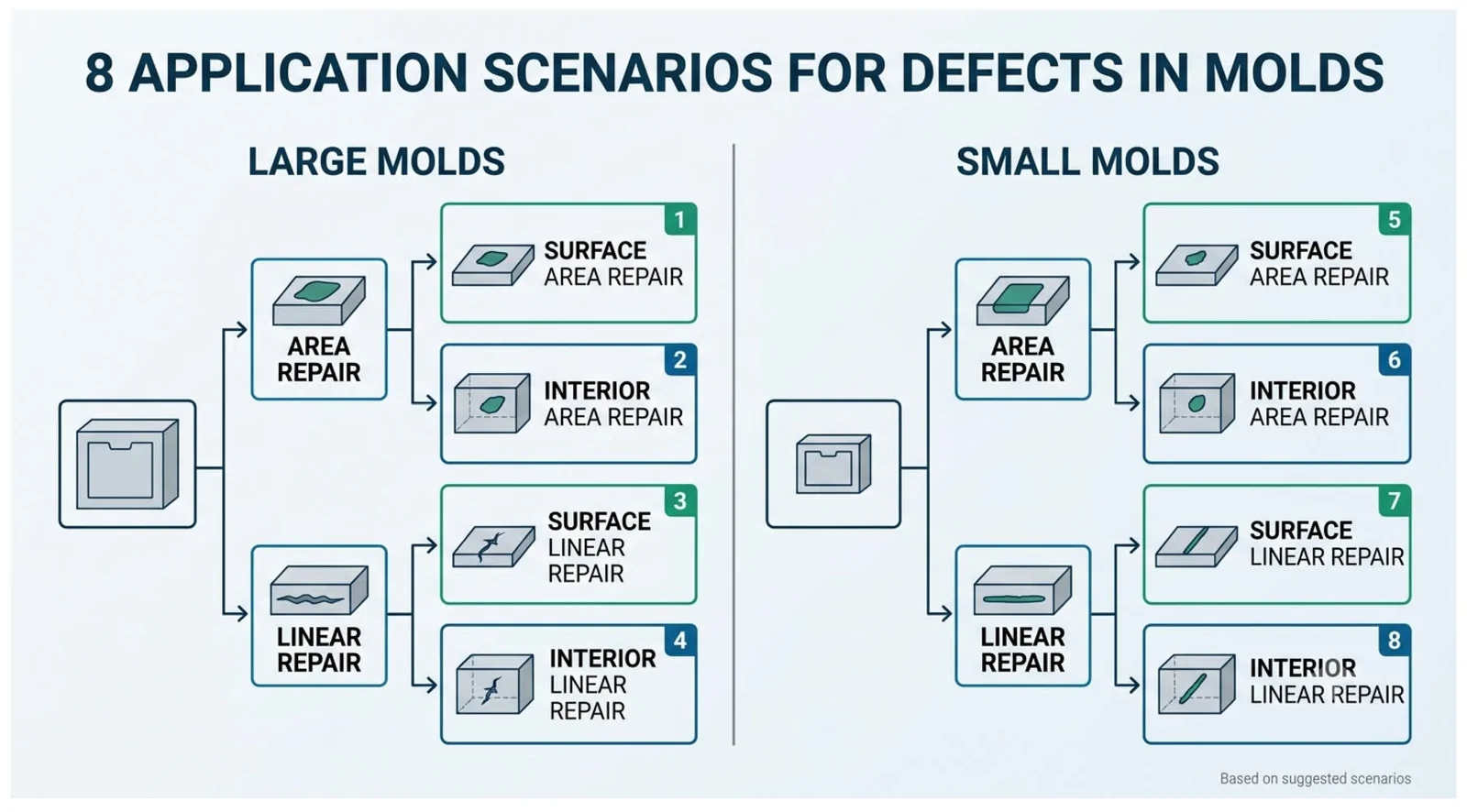
Eight application scenarios for defects in molds, adapted from [165].

Seeking to determine the most suitable process to repair molds of injection molding in different situations, Peças and Henriques [165] developed a comparison methodology where, firstly, the molds were split into small and large molds, being considered small those that weigh up to 2.5 tons. Small molds could be easily transported to repair shops where the welding equipment could repair the damaged mold; that way, equipment with low portability, such as a laser, still stands as an option. However, when the mold is large, it is advisable to move the welding equipment to where the mold is located; in this case, portability is important. The authors also classified the molds by their type of geometry to be repaired; according to them, there are two main types: linear geometry, which is related to the repair of edges, and area geometry, which is related to the repair of holes or rebuilding a feature. The reason for this distinction is that for edge repairs, the deposition rate is not relevant, as it is for area geometry repairs. The authors also described the repair in terms of a repair zone; there are two: the surface of the mold (surface) and the inner part of a depression of the mold (interior), so an important factor to be analyzed is whether the weld process is capable of accessing the interior of a complex geometry or if it is not relevant. Finally, the authors were able to suggest eight different application scenarios (see Figure 8), four for defects in large molds (area repair or linear repair), being area and linear repair split into surface or interior, and also linear repair split into surface and four for defects in small molds, following the same logic.

**Figure 9:**
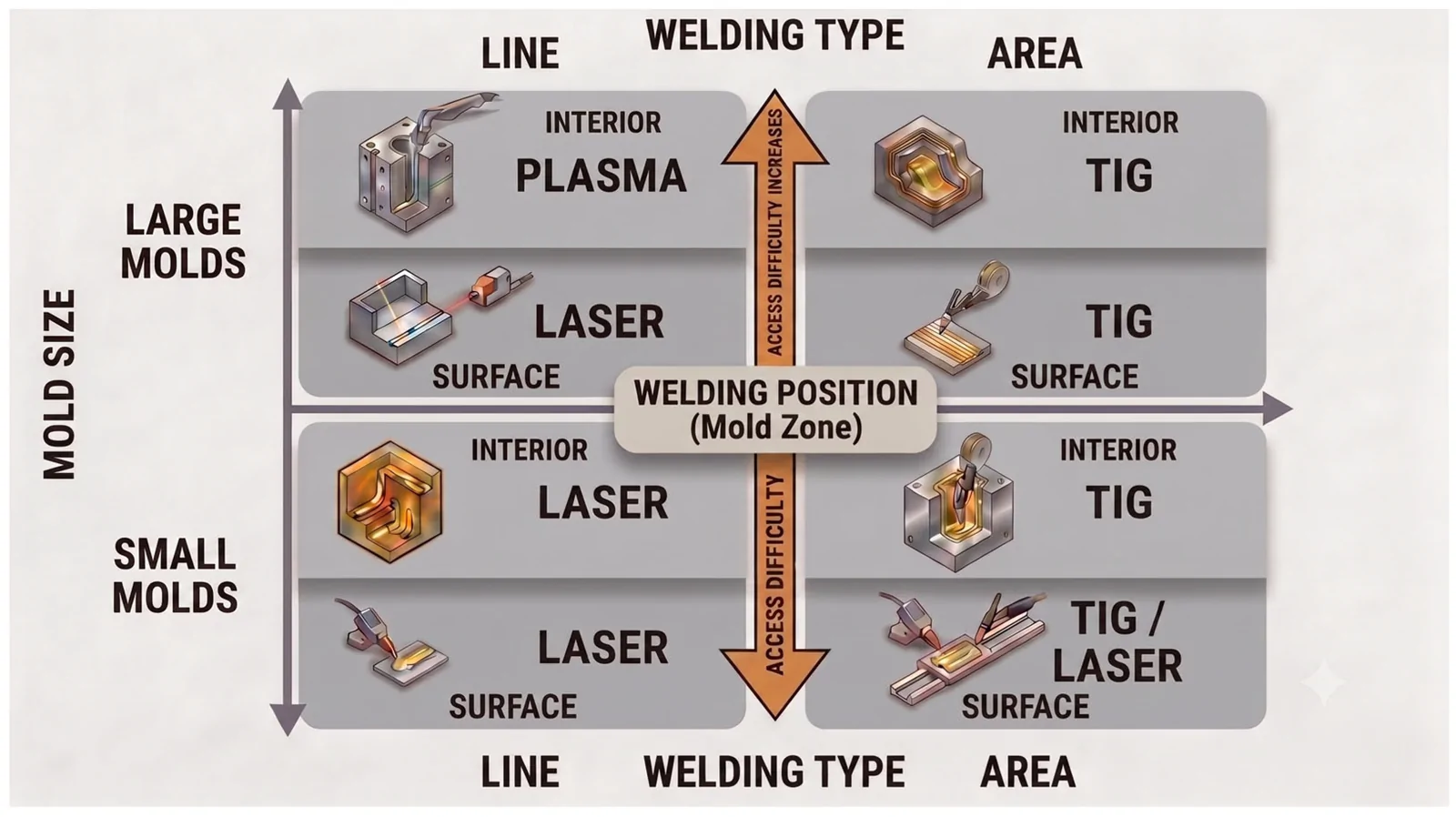
Most suitable process for each of eight scenarios, adapted from [165].

To identify the most suitable process for each scenario, the authors assign weights to each combination of evaluation criteria (e.g., deposition rate, portability, etc.) and repair zone. For example, the deposition rate in a surface area repair in a large mold (scenario 1) is relevant and receives the maximum weight. The weight of each combination, multiplied by the level of each process criterion and divided by the process with the best performance, yielded a relative performance parameter (%). The methodology delivered the most suitable process for each scenario, and one can see it more clearly in Figure 9.

Laser processes are the most preferable for line repairs, while GTAW is most recommended for area repairs. It is reasonable, especially for small molds, as laser is more accurate, with better metallurgical properties, and deposition rate is not relevant for linear repairs. The plasma process emerges as concurrent in large molds because, in this case, equipment portability matters, reaching 98% of performance in scenario 2, where laser still could keep the preference by its metallurgical quality and bead appearance; however, for scenario 1, plasma was preferable as interior repairs need equipment access to intricate geometries. Concerning area repairs, GTAW has taken the preference for large molds by its relatively high deposition rate and low cost, allied to good portability and geometry access. Laser becomes competitive in surface area repairs of small molds as deposition rate and portability are not more relevant. After this discussion, a question that remains is why PTAW is not recommended as GTAW is to repair large molds? The answer is deposition rate and cost. Regarding cost, GTAW is assumed to be unbeatable, but deposition rate can be surpassed by PTAW [166], either with wire or powder feed configuration [167]. The explanation that PTAW was considered to have a lower deposition rate than GTAW is probably because the review authors considered the manual hand-feed wire version of PTAW. Actually, the powder-feed plasma transferred arc welding (PTAW-P or PPTAW) is preferred over wire feed. The powder is easier to feed and requires lower heat input to melt [167].

PTAW-P is a promising process for hardfacing and repairing worn areas in parts such as gas turbine blades and vanes [168], marine diesel engines’ exhaust valves [169], agricultural equipment [170,171], and marble milling cutting tools [172].

Despite its application as the most well-known hardfacing process, it was possible to find reports in the literature of PTAW-P deposits on steel tool substrates, as mentioned above in Bibliometric Analysis, which would be a good approximation of its performance in mold and die repair. The AISI D2 was the most reported tool steel deposited by PTAW. Analyzing those reports could indicate that the D2 substrate pre-heating (around 300 °C) is essential to avoid thermal shock and crack initiation, as well as to retard the cooling rate of the base metal to reduce the crack susceptibility [133,135,137,138,141]. In the D2 tool steel study where WC composites were filler materials, the pre-heating could enable a more homogeneous distribution of primary carbides compared to the substrate that was not preheated [136].

From the abovementioned applications, allied with the equality in many aspects reported in Table 4, we can indicate the application of PTAW-P to repair molds and dies of relatively large volume.

The inherent fast cooling of the PTAW process often results in retained austenite in the matrix [152,154,155], so post-heat treatment is needed that promotes almost full transformation of retained austenite in martensite, increasing the hardness and wear resistance [152]. Post-heat treatment can also lead to fine secondary carbide precipitation in deposits of Stellite alloys on AISI H13 steel in the matrix [145]. During tempering, the formation of new carbides can lead to hardening tempering, which improves the thermal fatigue resistance of Stellite alloys deposited on H10 tool steel [148]. Post-heat treatment in AISI D2 above 900 °C promotes the austenitization and dissolution of primary carbides (Cr_23_C_6_), allowing secondary carbides (Cr_7_C_3_) to precipitate more dispersed after tempering [136,138]. In multilayer deposits, the reheating during the next layer deposition acts as cyclic heat treatment on previous layers, tempering the martensite previously formed and promoting stress relieving. These thermal cycles can also refine the previous dendritic microstructure [150]. Layers of high-speed steels and Ni-based alloys have been deposited on H11 substrate; the microscopy analysis reveals a permissible quantity of defects classified as rare pores. All three additive alloys were recommended for repair purposes, with the Ni-based alloy showing the best performance in tribological aspects [23].

AISI H13 steel was selected as the reference material to investigate the influence of the welding process on the component’s microstructure. This selection was prompted by the widespread use of this alloy in die fabrication and the significant volume of literature identified, as highlighted in the previous section. The studies presented in Table 5 were obtained from the Bibliometric Analysis and are illustrated in Figure 6. They were specifically chosen for providing microstructural data without the incorporation of additional particles. A noteworthy constraint in this comparison arises from the fact that the studies utilize substrates with diverse initial microstructures and highly varied processing parameters; consequently, the following analysis is qualitative. Initially, as previously discussed in Figure 5, it is observed that arc-based processes utilize filler metal in wire format, whereas laser-based processes employ metallic powders.

Generally, the investigations are not conducted on actual dies; rather, most studies utilize deposition on a flat plate to facilitate layer construction via additive manufacturing. While three specific studies utilize an annealed substrate [54,90,111], the remaining research focuses on quenched and tempered materials. All investigations proposing sequential material deposition identify the formation of a textured microstructure, as anticipated in additive manufacturing processes. It is crucial to recognize that material deposits intended for component recovery will never possess identical properties to those of the original substrate.

Among the studies referenced in Table 3, arc-based processes report HAZ extensions within the literature cited in Table 3; arc-based techniques demonstrate HAZ extensions typically ranging from 1 to 3 mm [108,115]. In contrast, studies employing laser-based processes indicate that the HAZ magnitude remains below 0.5 mm [54,76].

In the investigation conducted by Guzanová et al. [90], a comparative analysis between laser welding and GMAW for the restoration of AISI H13 steel was performed. Hardness measurements explicitly demonstrate that laser-based processing results in an abrupt hardness gradient between the deposited metal and the substrate, a direct consequence of a highly localized heat-affected zone. Conversely, the GMAW process exhibited a significantly greater extension of the affected area. Nevertheless, this variation does not appear to adversely influence the wear resistance of the deposits, as both techniques demonstrated comparable performance. The researchers concluded that while both methodologies are suitable for component recovery, laser-based techniques are preferable for smaller components, whereas arc processes are better suited for larger parts requiring higher material deposition rates [90].

Broadly, studies evaluating the wear characteristics of deposits observe performance at least equivalent to that of the base metal, irrespective of the energy source utilized [29,90,115]. This is attributed to the deposited material exhibiting higher hardness relative to the substrate. However, hardness alone does not dictate material performance; residual stresses, carbide distribution, and the profile of hardness variation across the cross-section are critical factors in determining the durability of a repair. Ultimately, the component is subjected to fatigue in addition to wear. Notably, none of the evaluated investigations conducted testing at the elevated temperatures where these materials are conventionally applied.

The microstructural analysis across the evaluated research reveals a predominantly martensitic matrix characterized by dispersed carbides and, in specific instances, the presence of retained austenite. It is important to emphasize that direct microstructural comparisons remain inherently constrained; beyond the variation in thermal cycles, the materials employed for both substrate and filler addition exhibit subtle discrepancies in their chemical compositions.

Discrepancies in thermal energy input can facilitate the precipitation of distinct carbides. Using the wire arc additive manufacturing technique, Du et al. [111] identified the predominant emergence of vanadium-rich carbides. Conversely, in the research conducted by Braga et al. [54] with the laser DED process, they identified carbides primarily composed of molybdenum and chromium. In the study conducted by Sun et al. [173], a comparative investigation was performed between laser beam welding (LBW) and GMAW using Q235 steel as the substrate. The researchers evaluated the thermal cycles for both methodologies, identifying a peak temperature of 2667 °C for the laser-based process, whereas the electric arc technique reached only 1722 °C. Furthermore, significant discrepancies in thermal history were noted, as the cooling rates associated with laser processing were considerably higher than those observed in the conventional arc process [173]. Sources for thermal discrepancies such as peak temperatures and exposure durations significantly influence the dissolution of alloying elements, thereby inducing distinct microstructural variations.

The development of retained austenite within the joint is further influenced by the peak temperature and subsequent cooling rates. Among the selected research, two studies employing electric arc energy sources identified the presence of this specific microconstituent [111,115], and only a single investigation utilizing laser-based energy sources identified this microconstituent within the joint [29]. Not all investigations prioritize identifying this microconstituent. However, its monitoring is fundamental, as in-service austenite may undergo transformation [111], inducing volumetric variations that facilitate crack nucleation.

Phase formation control can be executed through strategic adjustments to the thermal cycle, where variables such as heat input, welding speed, and preheating remain paramount. Within the literature, only three investigations utilized temperature control during material deposition, specifically through pre- or interpass heating, with all such instances employing electric arc energy sources [9,108,111]. Conversely, laser-based processes exhibit significantly higher cooling rates due to their capacity for lower heat input and elevated speeds. While the omission of preheating in these cases may seem counterintuitive, it is likely justified by the resulting reduction in the heat-affected zone. Notably, no studies reported the occurrence of welding-induced cracks. Furthermore, post-heating remains a fundamental practice for facilitating stress relief and phase control, particularly regarding the reduction in retained austenite; however, this stage was not conducted in any of the research presented in Table 4.

Upon reviewing the literature, it is evident that both energy sources—arc and laser—effectively process AISI H13 steel. Distinct methodologies successfully achieved deposits characterized by robust adhesion and a lack of defects. Notably, the laser-based process is distinguished by a reduced HAZ and minimal distortion, while demonstrating superior repair performance in both wear and corrosion evaluations [29,54,76]. Arc-based processes likewise yielded successful results, demonstrating properties that were at least comparable to those of the substrate material [29,76,111].

Plasma welding delivers high penetration as well as higher depth-to-width ratios, better temperature control, and a more restricted HAZ than conventional arc processes [166,174]. In the same vein, it was reported to have a higher deposition rate and high deposition efficiency, and it achieves thick overlay layers [166,170]. PTAW deposits high temperature tested exhibited superior microstructural stability, whereas laser deposits undergo significant changes [175]. Furthermore, it does not encounter processing difficulties when applied to materials with high surface reflectivity, a well-known limitation inherent to laser-based techniques [176].

## 5. Concluding Remarks

Repairing tools such as molds and dies can save important resources, since the supply for their manufacturing involves a long chain, including mining, steel production, forging, heat treatment, machining, and surface engineering.

It is essential to emphasize that the preceding discussions are primarily captured from the academic literature; however, component repair is extensively practiced across various industrial sectors. In industrial environments, the selection of a welding process is likely governed by organizational scale, equipment availability, and technical expertise.

Based on the summaries presented along with this review, we can draw forward the following conclusions:

- The most intriguing issue from the review literature is the scarcity of investigations applying PTAW to repair molds and tools. A hypothesis is that the number of platforms of this technique is much lower than those for the concurrent process, GTAW, which increases the initial cost.
- Despite the literature spanning the aspects attributed to PTAW, such as high deposition rate, high equipment portability, and high access and ability to repair complex geometries allied to a manageable cost, heat-treatment and metallurgical quality encourage the authors to suggest this process as a potential candidate for large mold surface or interior repair.
- Laser-based energy sources feature prominently in the literature, while electric arc techniques are less frequent. For both modalities, additive manufacturing strategies have been increasingly adopted for repair applications.
- High-quality, defect-free repairs with mechanical properties suitable for industrial requirements can be achieved regardless of the chosen energy source.
- The resulting microstructures are inherently linked to the welding thermal cycle, requiring precise control over heat input and deposition rate. Preheating and post-weld heat treatments are essential for managing phase formation and residual stresses.
- Most investigations lack application to real dies, and wear testing and microstructural analyses are often limited, complicating the assessment of long-term repair durability.

Assessing repair viability and selecting the most appropriate process are sophisticated undertakings. They necessitate profound technical knowledge to estimate the tool’s remaining useful life and optimize process parameters for an ideal microstructure.

## Figures and Tables

**Table 1:** Steel alloys commonly used to manufacture molds and tools for injection molding [15].

Material Number (DIN)	AISI (Equivalent)	Applications	Weldability
**Pre-Hardened Plastic Mold Steels**			
1.2311	P20	Compression and injection molds with a thickness of up to 400 mm.	Good
1.2312	P20 + S	Core components for compression and injection molds.	Conditionally suitable
1.2711	L6 mod.	Molds with higher mechanical and thermal stress.	Good
1.2738	P20 + Ni	Female mold for compression and injection molds of more than 400 mm.	Good
2738 mod. TS	P20 + Ni mod.	Large molds, female molds for compression, and injection molds of large dimensions.	Very good
**Through-Hardening Plastic Mold Steels**			
1.2343	H11	Highly stressed plastic molds, mold inserts with a high abrasive stress.	Neutral
1.2379	D2	Plastic molds and mold inserts with a particularly high resistance against wear.	Conditionally suitable
1.2767	6F7	Highly stressed mold, large molds for bodywork parts.	Good
1.2842	O2	Small plastic molds without specific requirements on polishability.	Conditionally suitable
**Corrosion-Resistant Plastic Mold Steels**			
1.2083	420	Small and mid-size molds and mold inserts, e.g., for PVC processing.	Good
1.2085	420F	Small and mid-size mold frames, e.g., for molds for PVC processing.	Good
1.2316	420 Mod.	Mold inserts, slit/profile dies, blow molds, mid-size and large molds.	Good
**Plastic Mold Steels for Case Hardening**			
1.2162	5120	Molds with compressive stress and abrasive surface load.	Good
1.2764	9310	Molds with high compressive stress, abrasive surface load, and high polishability.	Good
**Precipitation Hardening Plastic Mold Steels**			
1.2709	Maraging 300	Part inserts and small molds with high toughness requirements.	Very good
**Nitriding Steels**			
1.7735	4115 Mod.	Components for plastics processing with high frictional wear.	Neutral (need to find reference)
1.8519	4130 Mod.	Screw cylinders and barrel extruders.	Neutral
1.8550	Nitralloy N	Screw cylinders and barrel extruders requiring deep nitriding depth.	Neutral

**Table 2:** Most common forging die materials for manufacturing. Adapted from [17].

Material Number (DIN)	AISI (Equivalent)	Typical Applications
1.2713	6F2/L6	Hammer drop forging for small and medium sizes
1.2714	L6 (Mod.)	Hammer drop forging of large dimensions, complex engravings or inserts
1.2744	6F3	Forging dies for hammers
1.2343	H11	Conventional forging dies, tools for presses
1.2344	H13	Same as H11, but with higher hot wear resistance
1.2365	H10	Forging dies with good toughness for smaller parts
1.2367	H10 (Mod.)	Similar to 1.2365, but with higher thermal stability
1.2567	H21 (Low W)	Similar to 1.2365, but with less toughness

**Table 3:** Summary of studies on the PTA process with a focus on component repair.

Reference	Title	Year	Repaired Part
[127]	Cavitation Erosion Resistance of Deposits Processed by Plasma Transferred Arc Welding	2008	Bead on plate—hydraulic turbine
[128]	Cavitation Erosion Resistance of CrMnSiN Austenitic Stainless Steels Deposited by PTA	2010	Bead on plate—water pumps, valves and blades of hydraulic turbines
[13]	Plasma Transferred Arc Forming Technology for Remanufacture	2013	Thrust of engine cylinder
[129]	Development of Micro-Plasma Transferred Arc (Μ-Pta) Wire Deposition Process for Additive Layer Manufacturing Applications	2014	Bead on plate—dies and molds
[130]	Enhancement of Deposition Quality in Micro-plasma Transferred Arc Deposition Process	2014	Multi-layer for repair
[22]	Micro-Plasma Transferred Arc Additive Manufacturing for Die and Mold Surface Remanufacturing	2016	Bead on plate—dies and molds
[131]	Residual Stress Formation After Re-Contouring of Micro-Plasma Welded Ti-6Al-4V Parts by Means of Ball End Milling	2017	Bead on plate—compressor blade
[132]	Repair of Damaged Bearings Raceways by Means of Deposition Welding	2024	Bearings raceways

**Table 4:** Comparison among molds and die repair processes. Symbols refer to different authors: * [6], ** [162], *** [163].

Evaluation Criteria	Conventional TIG/GTAW	Plasma/PTAW	Micro-TIG/Micro-Plasma	Laser-Based Deposition
Deposition Rate	Good * Excellent ** Good ***	Good * Good ** Average ***	Poor *	Average * Poor ** Poor ***
Equipment Portability	Good * Excellent ** Good ***	Good * Good ** Good ***	Good *	Poor * Very poor ** Poor ***
Access and Ability to Repair Complex Geometries	Good * Good ** Excellent ***	Good * Good ** Good ***	Good *	Poor * Average ** Poor ***
Metallurgical Quality	Very poor * Average ** Very poor ***	Very poor * Average ** Poor ***	Average *	Good * Excellent ** Average ***
Post-Weld Heat Treatment	Very poor * Very poor ** Poor ***	Very poor * Average ** Poor ***	Average *	Good * Good ** Average ***
Low-cost	Excellent * Excellent ** Excellent ***	Excellent * Average ** Good ***	Poor *	Poor * Poor ** Poor ***
Low Setup Time	Good * Negligible **	Good * Negligible **	Average *	Poor * Average *

**Table 5:** Summary of research on AISI H13 steel as both base and filler metal for mold recovery applications.

Reference	Title	Year	Energy Source—Process	Metal Filler Type
[108]	Design and Testing of a WAAM Retrofit Kit for Repairing Operations on a Milling Machine	2021	Arc—WAAM GMAW	Wire
[111]	H13 Tool Steel Fabricated by Wire Arc Additive Manufacturing: Solidification Mode, Microstructure Evolution Mechanism and Mechanical Properties	2023	Arc—WAAM GMAW	Flux-cored wire
[9]	Life-Cycle Energy and Carbon Saving Potential of Wire Arc Additive Manufacturing for the Repair of Mold Inserts	2021	Arc—WAAM GMAW	Wire
[115]	Numerical Simulation of Thermal Field and Performance Study on H13 Die Steel-Based Wire Arc Additive Manufacturing	2023	Arc—WAAM GTAW	Wire
[54]	Microstructural and Mechanical Aspects of Laser Metal Deposited H13 Powder for Die Repair	2021	Laser—Laser Metal Deposition	Powder
[76]	Anisotropic Corrosion Behavior of Laser-Based Direct Energy Deposited H13 Steel in Molten Adc12 Aluminum Alloy	2024	Laser—Laser DED	Powder
[90]	The Corrosion and Wear Resistance of Laser and Mag Weld Deposits	2020	Arc—GMAW and Laser Welding	Wire
[29]	Wear Characteristics of Std61 Tool Steel According to Repairing Methods for Al Port-Hole Extrusion Die: Direct Metal Deposition, Welding, Parent Material	2018	Arc—GMAW and Laser—Direct Metal Deposition	Wire and Powder

## Data Availability

No new data were created or analyzed in this study. Data sharing is not applicable to this article.

## Abbreviations

The following abbreviations are used in this manuscript:

µ-GTAW	Micro-Gas Tungsten Arc Welding
µPTAW	Micro-Plasma Transferred Arc Welding
Arc-DED	Arc Directed Energy Deposition
DMD	Direct Metal Deposition
DMLS	Direct Metal Laser Sintering
GMAW	Gas Metal Arc Welding
GTAW	Gas Tungsten Arc Welding
HAZ	Heat-Affected Zone
Laser-DED	Laser-Directed Energy Deposition
LBW	Laser Beam Welding
LCA	Life Cycle Assessment
PPTAW	Powder-Feed Plasma Transferred Arc Welding
PTA	Plasma Transferred Arc
PTAW	Plasma Transferred Arc Welding
PTAW-P	Powder-Feed Plasma Transferred Arc Welding
SAW	Submerged Arc Welding
SMAW	Shielded Metal Arc Welding
SMEs	Small and Medium-Sized Enterprises
TIG	Tungsten Inert Gas
WAAM	Wire Arc Additive Manufacturing
